# Depression-Like Effect of Prenatal Buprenorphine Exposure in Rats

**DOI:** 10.1371/journal.pone.0082262

**Published:** 2013-12-18

**Authors:** Chih-Jen Hung, Chih-Cheng Wu, Wen-Ying Chen, Cheng-Yi Chang, Yu-Hsiang Kuan, Hung-Chuan Pan, Su-Lan Liao, Chun-Jung Chen

**Affiliations:** 1 Department of Anesthesiology, Taichung Veterans General Hospital, Taichung, Taiwan, ROC; 2 Graduate School of Nursing, HungKuang University, Taichung, Taiwan, ROC; 3 Department of Financial and Computational Mathematics, Providence University, Taichung, Taiwan, ROC; 4 Department of Veterinary Medicine, National Chung Hsing University, Taichung, Taiwan, ROC; 5 Department of Surgery, Fong Yuan Hospital, Taichung, Taiwan, ROC; 6 Department of Pharmacology, Chung Shan Medical University, Taichung, Taiwan, ROC; 7 Department of Neurosurgery, Research, Taichung Veterans General Hospital, Taichung, Taiwan, ROC; 8 Faculty of Medicine, School of Medicine, National Yang-Ming University, Taipei, Taiwan, ROC; 9 Department of Education and Research, Taichung Veterans General Hospital, Taichung, Taiwan, ROC; 10 Institute of Biomedical Sciences, National Chung Hsing University, Taichung, Taiwan, ROC; 11 Center for General Education, Tunghai University, Taichung, Taiwan, ROC; Indian Institute of Toxicology Reserach, India

## Abstract

Studies indicate that perinatal opioid exposure produces a variety of short- and long-term neurobehavioral consequences. However, the precise modes of action are incompletely understood. Buprenorphine, a mixed agonist/antagonist at the opioid receptors, is currently being used in clinical trials for managing pregnant opioid addicts. This study provides evidence of depression-like consequence following prenatal exposure to supra-therapeutic dose of buprenorphine and sheds light on potential mechanisms of action in a rat model involving administration of intraperitoneal injection to pregnant Sprague-Dawley rats starting from gestation day 7 and lasting for 14 days. Results showed that pups at postnatal day 21 but not the dams had worse parameters of depression-like neurobehaviors using a forced swimming test and tail suspension test, independent of gender. Neurobehavioral changes were accompanied by elevation of oxidative stress, reduction of plasma levels of brain-derived neurotrophic factor (BDNF) and serotonin, and attenuation of tropomyosin-related kinase receptor type B (TrkB) phosphorylation, extracellular signal-regulated kinase (ERK) phosphorylation, protein kinase A activity, cAMP response element-binding protein (CREB) phosphorylation, and CREB DNA-binding activity. Since BDNF/serotonin and CREB signaling could orchestrate a positive feedback loop, our findings suggest that the induction of oxidative stress, reduction of BDNF and serotonin expression, and attenuation of CREB signaling induced by prenatal exposure to supra-therapeutic dose of buprenorphine provide evidence of potential mechanism for the development of depression-like neurobehavior.

## Introduction

Maintenance treatment with methadone is the current recommended standard of care for opioid-dependent pregnant women, even though the µ-opioid receptor agonist has been shown to predispose the infants to develop symptoms of neonatal abstinence syndrome which is characterized by autonomic and central nervous system hyperactivity, often with associated gastrointestinal tract and respiratory system dysfunction [1], [2]. Buprenorphine, a semisynthetic opioid derivative, which acts as a partial agonist at the µ-opioid receptor and as an antagonist at κ- and δ-opioid receptors [3], was approved by the U.S. Food and Drug Administration in 2002 for the management of opioid dependence in non-pregnant patients, although it has been available for many years to treat pain. Currently, this drug is undergoing clinical trials for the management of pregnant opioid addicts. Accumulating evidence suggests that buprenorphine may offer advantages over the use of methadone such as reduced severity of neonatal abstinence syndrome [4]–[7].

Research from human and animal studies has raised concerns regarding the potential adverse effects of methadone and buprenorphine treatments. Acute buprenorphine exposure induces locomotor stimulation and conditioned place preference [8]. Of particular note, perinatal opioid exposure has been demonstrated to produce a variety of short- and long-term neurobehavioral consequences in offspring [7], [9]–[11]. Experimental studies have further shown that perinatal exposure to buprenorphine affects neurotrophic factor and neurotransmitter biosynthesis, neurogenesis, and myelination [12]–[15]. In spite of the well-known neurobehavioral effects of buprenorphine, the molecular and cellular basis underlying its mechanisms of action are incompletely understood.

All opioids taken by the mother are highly lipophilic, easily passing the blood-brain and placental barrier alike, exposing the developing brain of the fetus to unfavorable effects, although the complexity of these mechanisms is not fully understood [16]. Buprenorphine represents an opioid with a unique and complex pharmacology because it can simultaneously act as an agonist and/or antagonist at different classes of opioid receptors and has a high affinity for and slow association and dissociation from receptors, which results in a long duration of action [17]. Since neural cells express opioid receptors, these pharmacological characteristics of buprenorphine highlight its potential involvement in the development of fetal brain and neurobehavioral consequences. Evidence shows that depression is common among opioid-dependent patients and is associated with a poor prognosis [18]. However, the potential development of depression-like neurobehavior after perinatal opioid exposure is still not reported. In view of the current clinical trial which is underway for maintenance treatment of pregnant addicts, further research on the neurobehavioral effects of this drug during pregnancy is urgently needed. To extend the scope of relevant studies, we therefore undertook the present investigation to examine the potential depression-like effect of prenatal buprenorphine exposure in weanlings and identify causative mediators involved.

## Materials and Methods

### Animals and Buprenorphine Treatment

The Animal Experimental Committee of Taichung Veterans General Hospital approved the protocol of the animal study (La-99747). All efforts were made to minimize animal suffering and to reduce the number of animals used, if available. Female Sprague-Dawley rats (200–250 g) were housed for at least 1 week in their home cages at a constant temperature, with a 12-hour light-dark cycle, and *ad libitum* access to food and water. Females were placed individually with male conspecifics during breeding. The detection of vaginal sperm plug was used to indicate successful mating and was defined as gestation day 0. After confirmation of mating, female rats (60 animals) were randomly allocated into three experimental groups (n = 20 per group). On day 7 of gestation, these pregnant rats started to receive daily (9∶00 AM) single intraperitoneal injection of buprenorphine (0, 0.3, or 1 mg/kg, Unichem Bhavan, Mumbai, India) for 14 days. The experimental protocols and buprenorphine dosages were conducted and modified from relevant reports [13], [14]. After birth, the litters were kept separate. On postnatal day 21, the weanlings and dams were collected and subjected to further analyses. Consequent analyses were done by randomly selecting 1 male and 1 female pup from each little for each assay.

### Behavioral Observations

The modified open field test, Morris water maze, forced swimming test, and tail suspension test were conducted according to previously reported methods with some modifications by a technician blinded to the treatments [19], [20]. For the measurement of spontaneous locomotion, animals were placed on the apparatus (30 cm×30 cm×30 cm) for the next 20 min and the travel distance and moving time were recorded. In Morris water maze, the task for all of the animals in each trial consisted of finding a hidden platform (10 cm diameter) that was placed 50 cm away from the wall of the water maze (150 cm in diameter, 60 cm in depth) and 1 cm below the water. The time required to reach the hidden platform was recorded. The animals were allowed to rest 30 s on the platform between trials. Each animal underwent three sessions per day for three consecutive days. For the forced swimming test, animals were individually placed in a glass cylinder (50 cm in height and 20 cm in diameter) filled with water to a depth of 30 cm (24±2°C). All animals were forced to swim for 5 min and the duration of behavioral parameters were recorded in seconds. The behavioral parameters included immobility time, climbing time, and swimming time. For the conduction of tail suspension test, animals were individually suspended by the tail with a clamp (1 cm from the tip of the end) in a box (25 cm×25 cm×30 cm) with the head 5 cm from the bottom. All animals were suspended for 6 min and the duration of behavioral parameters including immobility time and climbing time were recorded in seconds. To evaluate the effect of antidepressant, imipramine (20 mg/kg, Tocris Bioscience, Bristol, UK) was administrated intraperitoneally 1 h prior to test in accordance with previously reported protocols [19].

### Measurement of Lipid Peroxidation

A thiobarbituric acid reactive substances (TBARS) assay kit (ZeptoMetrix, Buffalo, NY, USA) was used to measure the lipid peroxidation products, malondialdehyde (MDA) equivalents. In brief, cortical tissues were homogenized with 0.1 mol/l sodium phosphate buffer (pH 7.4). One hundred microliters of homogenate were mixed with 2.5 ml reaction buffer (provided in the kit) and heated at 95°C for 60 min. After the mixture had cooled, the absorbance of the supernatant was measured at 532 nm using a spectrophotometer. The lipid peroxidation products are expressed in terms of MDA equivalents.

### Measurement of Glutathione Content

GSH and GSSG were determined using a commercially available glutathione assay kit (Cayman, Ann Arbor, MI, USA). Briefly, cortical tissues were weighed and homogenized with 0.1 M sodium phosphate buffer (pH 7.4). The homogenates were then centrifuged with 5% trichloroacetic acid to remove the proteins. An aliquot of 50 µl of homogenate was mixed with 150 µl reaction buffer (provided in kit). The mixture was vortexed and the absorbance read at 405 nm within 30 min. The content was calculated using a standard solution of GSH.

### Measurement of Antioxidant Enzyme Activity

Catalase and glutathione peroxidase (GPx) activities were determined using commercially available assay kits (Cayman, Ann Arbor, MI, USA). Briefly, cortical tissues were weighed and homogenized with appropriate buffers (provided in the kits). The specific activities of the various enzymes are expressed in nmole/mg of the protein with the protein content determined as stated above.

### Measurement of Brain-derived Neurotrophic Factor (BDNF) and Serotonin Content

After decapitation, blood samples were withdrawn from femoral arteries. Plasma samples were stored at −20°C until assays were performed. Plasma levels of BDNF and serotonin were measured using an enzyme-linked immunosorbent assay (ELISA) (R&D Systems, Minneapolis, MN).

### Western Blot

Total proteins were extracted from cortical tissues using tissue protein extraction reagents (T-PER, Pierce Biotechnology, Rockford, IL). Protein extracts were resolved by SDS-polyacrylamide gel electrophoresis, and transferred onto a blotting membrane. The membranes were incubated with antibodies against tropomyosin-related kinase receptor type B (TrkB), phosphorylated TrkB, extracellular signal-regulated kinase (ERK), phosphorylated ERK (Santa Cruz Biotechnology, Santa Cruz, CA), cAMP response element-binding protein (CREB), phosphorylated CREB (Epitomics, Burlingame, CA), and β-tubulin (BD, San Diego, CA). Then, the membranes were incubated with horseradish peroxidase-conjugated secondary antibody. Specific protein bands were visualized by enhanced chemiluminescence. All measured protein levels were quantified by densitometry using a computer image analysis system (Alpha Innotech Corporation, IS1000, San Leandro, CA).

### Preparation of Nuclear Extracts and Electrophoretic Mobility Shift Assay (EMSA)

Nuclear extracts were prepared from cortical tissues. Briefly, the obtained cell nuclei were resuspended and lysed with extraction buffer (20 mM HEPES, pH 8.0; 420 mM NaCl; 1.5 mM MgCl_2_; 0.2 mM EDTA; 1 mM dithiothreitol; 10% glycerol; 0.5 mM phenylmethylsulfonyl fluoride; 1 mM NaF; 1 mM Na_3_VO_4_). The oligonucleotide of CREB (5′-AGAGATTGCCTGACGTCAGAGAGCTAG) was synthesized and 5′ labeled with biotin according to the recommendations of the manufacturer (Panomics, Fremont, CA). Nuclear extract (5 µg) was used for EMSA. The binding reaction mixture included 1 µg of poly (dI-dC), 0.1 µg of poly L-lysine, and 100 fmole of biotin-labeled DNA probe in 20 µl of binding buffer (10 mM HEPES, pH 7.6; 50 mM NaCl; 0.5 mM MgCl_2_; 0.5 mM EDTA; 1 mM dithiothreitol; 5% glycerol). The DNA/protein complex was analyzed on 6% native polyacrylamide gels and electrically transferred to nylon membranes. The labeled oligonucleotides were reacted with horseradish peroxidase-labeled streptavidin and detected using chemiluminescence reagents.

### Measurement of Protein Kinase A (PKA) Activity

In brief, cortical tissues were homogenized with 0.1 mol/l sodium phosphate buffer (pH 7.4). The measurement of PKA activity was performed using a commercially available PKA kinase activity assay kit (Assay Designs, Ann Arbor, MI) according to the manufacturer’s instructions.

### Statistical Analysis

The data are expressed as mean values ± standard deviation. Statistical differences between group means were evaluated by one-way analysis of variance (ANOVA), followed by Dunnett’s test. A level of p<0.05 was considered statistically significant.

## Results

### Prenatal Buprenorphine Exposure Caused Depression-like Neurobehavior

**Figure 1:**
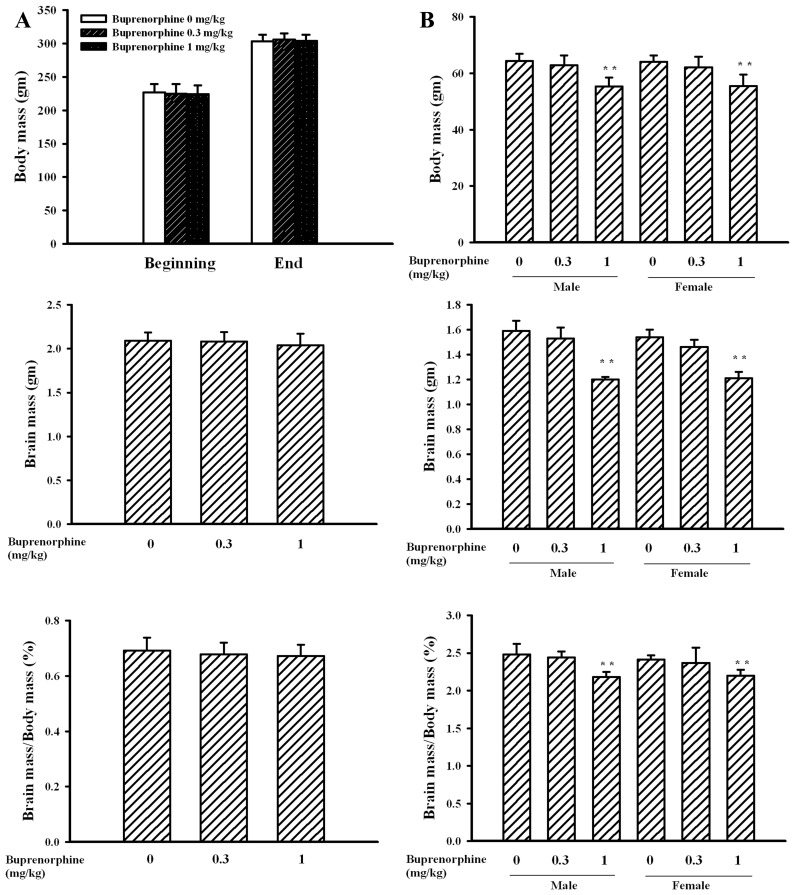
Buprenorphine decreased organ mass in pups. Prenatal buprenorphine (0, 0.3, and 1 mg/kg/day) exposure was started from gestation day 7 and lasted for 14 days. After birth, the male and female pups were collected at postnatal day 21. At this time, the body mass and brain mass of dams (A) and pups (B) were weighed and the ratio of brain mass in body mass (%) was calculated. The body mass of dams was recorded prior to the start of experiments (beginning) and at the end of experiments (end). **p<0.01 vs. each vehicle group, n = 20 per group.

Perinatal buprenorphine (0–3 mg/kg/day) exposure has been widely used to investigate its impact on rat pups from postnatal days 0 to 21. Studies have also revealed that a buprenorphine dose of 0.3 mg/kg/day is a level comparable to that used for the management of pregnant opioid addicts, and that 1 mg/kg/day is a dose equivalent to overexposure level [13]–[15]. To substantiate reported findings and further demonstrate the neurobehavioral effects of buprenorphine, pregnant rats were intraperitoneally administered with buprenorphine (0, 0.3, and 1 mg/kg/day) starting from gestation day 7 and lasting for 14 days, and the evaluation of pups was conducted on postnatal day 21. During the course of this study, data of dams’ food intake and water intake were not significantly different among groups. None of the treatments significantly affected the sizes of litters, the numbers of live pups born, and the numbers of male and female pups (data not shown). At the end of this study (∼6 weeks), there was no difference of body mass, brain mass, and percentage brain mass in the whole body in dams among groups (Fig. 1A). However, these parameters were lower in pups born to mothers receiving 1 mg/kg/day buprenorphine (Fig. 1B). These findings suggest that a prenatal supra-therapeutic dose of buprenorphine might impair organ development, particularly the brain, in weanlings.

**Figure 2:**
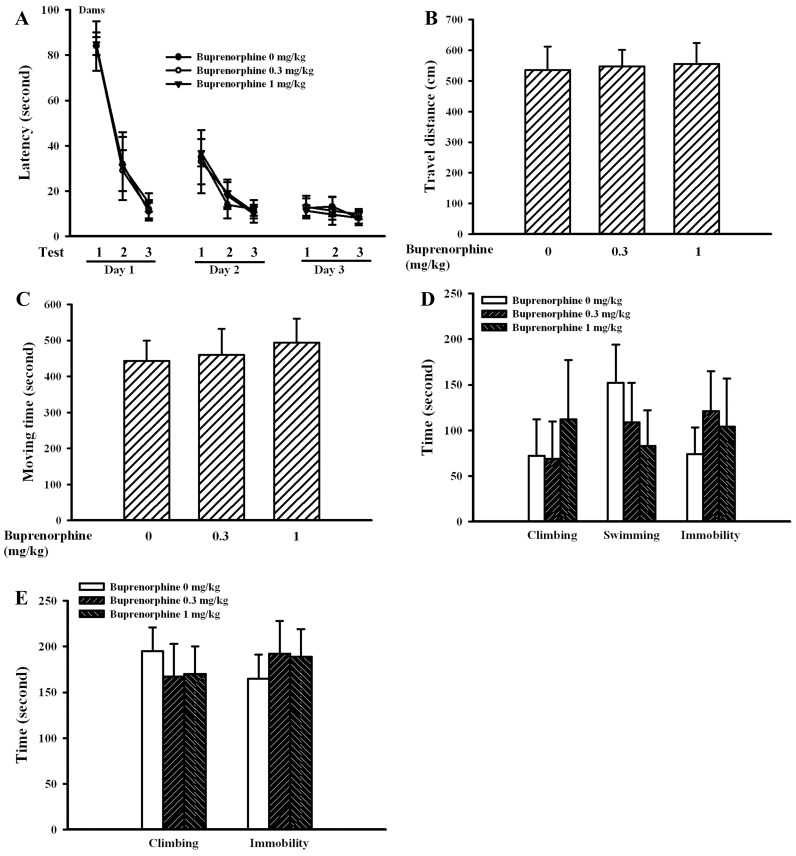
Buprenorphine had a negligible effect on neurobehaviors in dams. Prenatal buprenorphine (0, 0.3, and 1 mg/kg/day) exposure was started from gestation day 7 and lasted for 14 days. Three weeks after parturition, the dams were subjected to neurobehavioral test. The time required to reach the hidden platform (latency) was recorded in Morris water maze task (n = 5 per group). Each animal underwent three sessions (test 1–3) per day for three consecutive days (A). The spontaneous locomotion (n = 5 per group), including travel distance (B) and moving time (C), was measured for a period of 20 min using open field test. Forced swimming test (n = 5 per group) was conducted for a period of 5 min and the climbing time, swimming time, and immobility time were recorded (D). Tail suspension test (n = 5 per group) was performed for a period of 6 min and the climbing time and immobility time were recorded (E).

**Figure 3:**
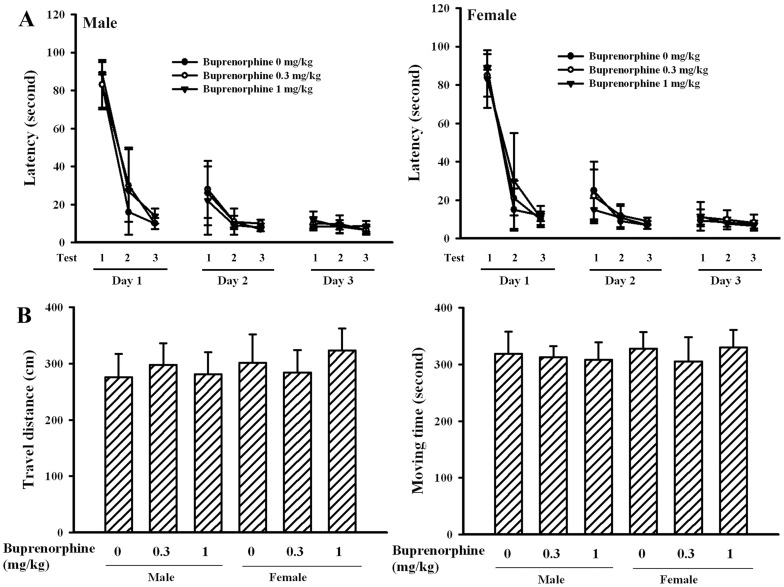
Buprenorphine had negligible effects on learning/memory and locomotor activity in pups. Prenatal buprenorphine (0, 0.3, and 1 mg/kg/day) exposure was started from gestation day 7 and lasted for 14 days. After birth, the male and female pups were collected at postnatal day 21 and subjected to neurobehavioral test. The time required to reach the hidden platform (latency) was recorded in Morris water maze task (n = 6 per group). Each animal underwent three sessions (test 1–3) per day for three consecutive days (A). The spontaneous locomotion (n = 6 per group), including travel distance and moving time, was measured for a period of 20 min using open field test (B).

**Figure 4:**
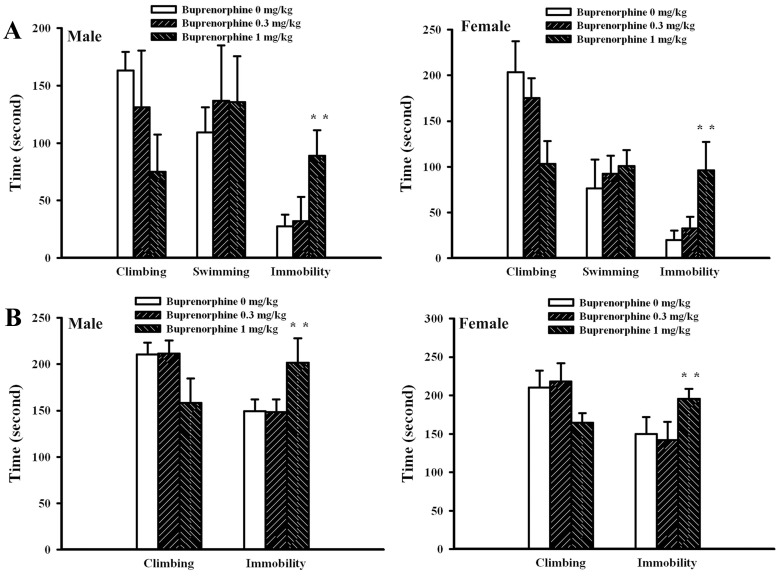
Buprenorphine induced depression-like neurobehaviors in pups. Prenatal buprenorphine (0, 0.3, and 1 mg/kg/day) exposure was started from gestation day 7 and lasted for 14 days. After birth, the male and female pups were collected at postnatal day 21 and subjected to neurobehavioral test. Forced swimming test (n = 6 per group) was conducted for a period of 5 min and the climbing time, swimming time, and immobility time were recorded (A). Tail suspension test (n = 6 per group) was performed for a period of 6 min and the climbing time and immobility time were recorded (B). **p<0.01 vs. each vehicle group.

**Figure 5:**
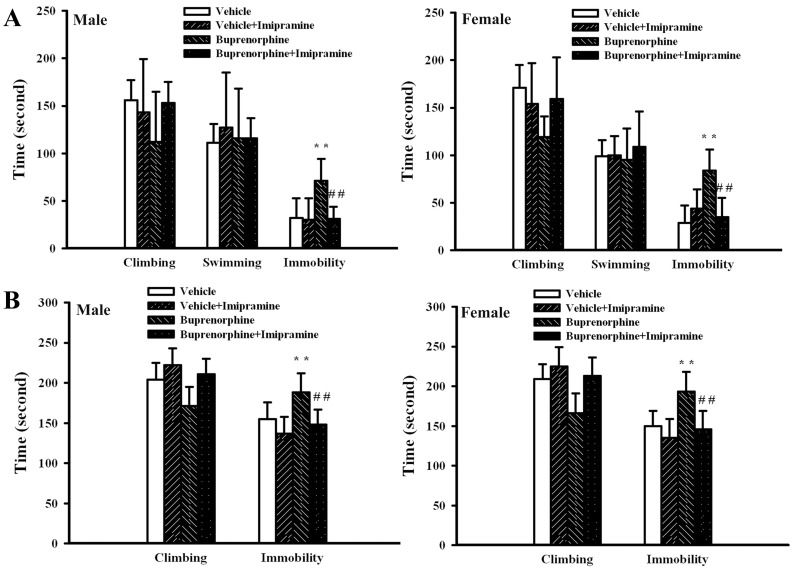
Imipramine attenuated buprenorphine-induced depression-like neurobehaviors in pups. Prenatal buprenorphine (0 and 1 mg/kg/day) exposure was started from gestation day 7 and lasted for 14 days. After birth, the male and female pups were collected at postnatal day 21 and subjected to neurobehavioral test. Male and female pups (n = 6 per group) were subjected to intraperitoneal imipramine (0 and 20 mg/kg) administration 1 h before forced swimming test (A) and tail suspension test (B). **p<0.01 vs. each vehicle group and ##p<0.01 vs. each buprenorphine group.

Generally, the Morris water maze task, open field test, forced swimming test, and tail suspension test were routinely used to evaluate neurobehaviors in animals [19]–[21]. Data of the time required to reach the platform in Morris water maze task (Fig. 2A), the travel distance (Fig. 2B) and moving time (Fig. 2C) in open field test, the immobility time in forced swimming test (Fig. 2D), and the immobility time in tail suspension test (Fig. 2E) showed that prenatal buprenorphine exposure did not cause significant neurobehavioral alterations in dams. Figure 3A and 3B show that the data of Morris water maze task and open field test were also not significantly different among groups in pups. However, a remarkable increase in immobility time for the forced swimming test (Fig. 4A) and tail suspension test (Fig. 4B) was found in the groups that received a supra-therapeutic dose of buprenorphine, independent of gender. To further demonstrate the potential for the development of depression-like neurobehavior in the groups that received a supra-therapeutic dose of buprenorphine, tricyclic antidepressant imipramine was intraperitoneally administered 1 h prior to neurobehavioral evaluation [19]. Imipramine had a negligible effect on the immobility time of the vehicle control group, whereas it caused a reduction in immobility time of the group that received a supra-therapeutic dose of buprenorphine in the forced swimming test (Fig. 5A) and tail suspension test (Fig. 5B). The results of the neurobehavioral evaluation suggest that prenatal supra-therapeutic dose of buprenorphine might lead to development of depression-like phenotypes in weanlings but not dams. Previous studies suggest a crucial role of biochemical changes in the prefrontal cortex, hippocampus, and amygdala for the development of depression-like neurobehavior [22], [23]. In this study, the cortical tissues were used for consequent biochemical analyses.

### Prenatal Buprenorphine Exposure Induced Oxidative Stress

**Figure 6:**
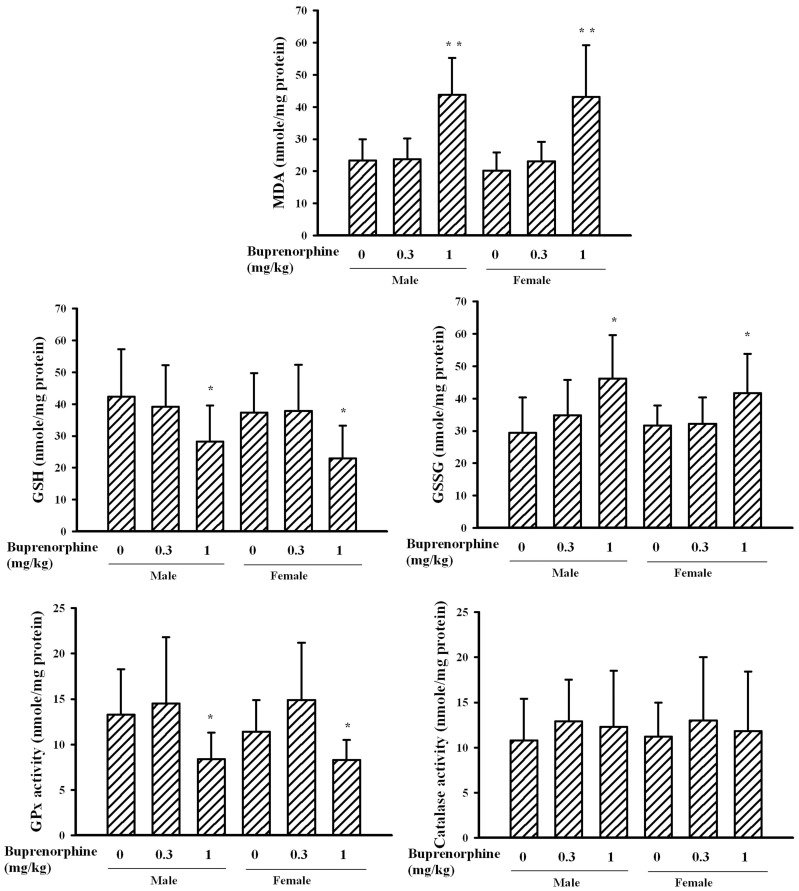
Buprenorphine altered oxidative potential. Prenatal buprenorphine (0, 0.3, and 1 mg/kg/day) exposure was started from gestation day 7 and lasted for 14 days. After birth, the male and female pups were collected at postnatal day 21. Brain cortical tissues were isolated and subjected to measurement of MDA content (n = 6 per group), GSH content (n = 5 per group), GSSG content (n = 5 per group), GPx activity (n = 5 per group), and catalase activity (n = 5 per group). *p<0.05 and **p<0.01 vs. each vehicle group.

It has been postulated that oxidative stress plays an important role in the pathogenesis of depression and responses to treatment [24], [25]. To further demonstrate the development of changes associated with depression-like neurobehavior, several parameters of oxidative stress in cortical tissues were determined. The level of lipid peroxidation product MDA, an index of oxidative stress, was elevated in male and female pups exposed to a supra-therapeutic dose of buprenorphine (Fig. 6). The changes in the levels of antioxidants and activity of anti-oxidative enzymes were inversely related to the increase in MDA levels. The cortical reduced GSH content and GPx activity were decreased in the groups that received a supra-therapeutic dose of buprenorphine compared to those of the vehicle control group. In contrast, a supra-therapeutic dose of buprenorphine caused a moderate elevation in oxidized GSH content and left the catalase activity unchanged. These data suggest that a prenatal supra-therapeutic dose of buprenorphine causes brain oxidative stress in pups of both genders.

### Prenatal Buprenorphine Exposure Caused a Reduction of Neurotrophins and Neurotransmitters

**Figure 7:**
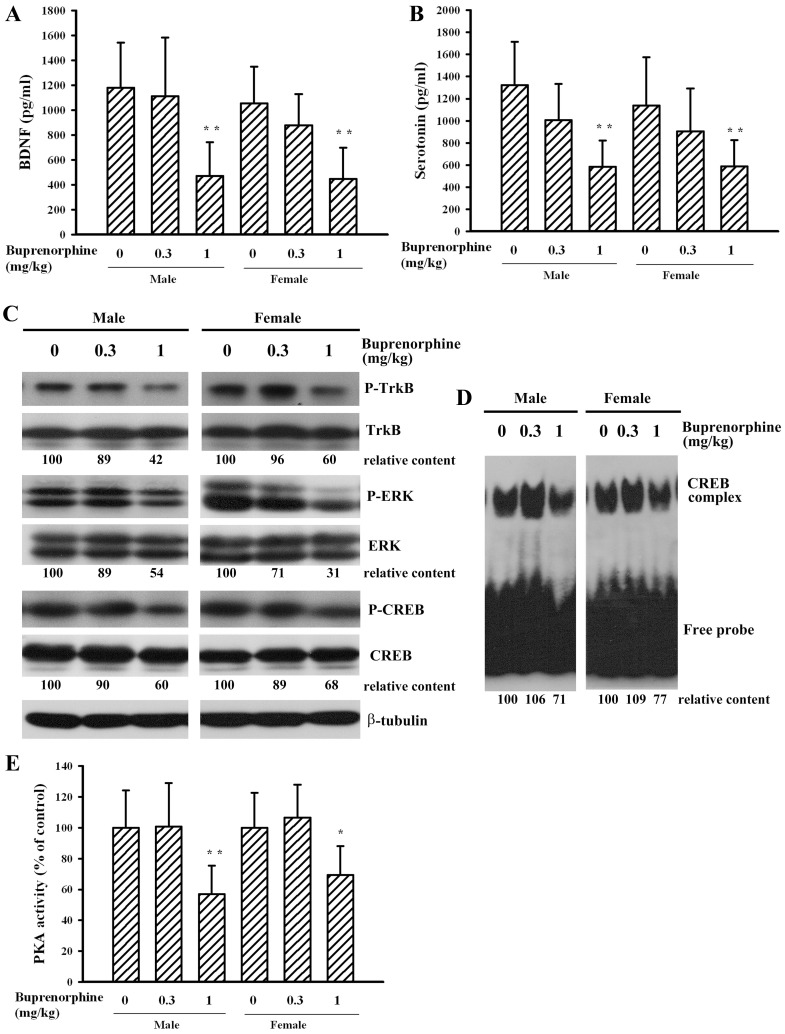
Buprenorphine decreased plasma BDNF and serotonin content and altered signaling molecule expression. Prenatal buprenorphine (0, 0.3, and 1 mg/kg/day) exposure was started from gestation day 7 and lasted for 14 days. After birth, the male and female pups were collected at postnatal day 21. Blood samples (n = 20 per group) were collected and subjected to ELISA for the measurement of BDNF (A) and serotonin (B). Brain cortical tissues were isolated. The obtained protein extracts (n = 5 per group) were subjected to Western blot analysis for the measurement of phosphorylated TrkB, TrkB, phosphorylated ERK, ERK, phosphorylated CREB, CREB, and β-tubulin (C). The obtained nuclear extracts (n = 5 per group) were subjected to EMSA for the measurement of CREB DNA binding activity (D). The protein extracts (n = 7 per group) were subjected to the enzymatic measurement of PKA activity (E). *p<0.05 and **p<0.01 vs. each vehicle group.

Accumulating evidence suggests that impaired BDNF signaling or disturbed serotonergic neurotransmission is the key mechanism in the pathophysiology of depression [23], [26]. Therefore, plasma levels in BDNF and serotonin were measured. Pups born to mothers treated with a supra-therapeutic dose of buprenorphine had decreased plasma levels of BDNF (Fig. 7A) and serotonin (Fig. 7B) in both genders compared with those found in pups born to mothers in the vehicle control group. These findings suggest that prenatal supra-therapeutic dose of buprenorphine has an inhibitory effect on circulating levels of BDNF and serotonin in weanlings.

### Prenatal Buprenorphine Exposure Impaired Signaling Molecules

CREB is one of the most important transcription factors for the expression of neurotrophins and downstream effectors of neurotrophins, and has also been shown to play an important role in depression [22], [23]. The depression-like effect of prenatal supra-therapeutic dose of buprenorphine was further investigated by examining its effect on CREB activity and intracellular signaling molecules critical to CREB activation. Supra-therapeutic dose of buprenorphine caused a significant decrease in the phosphorylation (Fig. 7C, p<0.01) and DNA-binding activity (Fig. 7D, p<0.01) of CREB in pups of both genders. The status of CREB phosphorylation is controlled by multiple upstream regulators, including ERK and PKA [22], [27], [28]. The results of Western blot and enzymatic assay revealed that supra-therapeutic dose of buprenorphine caused a reduction in ERK phosphorylation (Fig. 7C, p<0.01) and PKA activity (Fig. 7E). TrkB is a high-affinity and determinant membrane receptor crucial to BDNF signaling [21], [29]. Similarly, in the present study, a reduction of TrkB phosphorylation in the groups that received a supra-therapeutic dose of buprenorphine was found (Fig. 7C, p<0.01). These results suggest that pups of a dam exposed to a supra-therapeutic dose of buprenorphine showed a reduction in BDNF signaling and CREB activation.

## Discussion

Buprenorphine, a long-acting mixed agonist/antagonist of the opioid receptors, is an office-based maintenance treatment for opioid dependence. There is growing evidence that buprenorphine may serve as a reasonable alternative medication in pregnancy [4]–[7]. In the present study, although prenatal exposure to supra-therapeutic dose of buprenorphine had a negligible effect on maternal and fetal or neonatal mortality, we found that it decreased brain mass and body mass and caused a significant impact on depression-like neurobehavior in weanlings. Forced swimming test and tail suspension test are currently widely used paradigms to assess depression and anti-depression phenotypes. In these two tests, duration of immobility, a posture thought to reflect a state of behavioral despair in which animals have given up the hope of escape, is thought to be related to depression, and drugs with anti-depressant activity reduce the time that the animals remain immobile [19], [21], [30]. Together with the reversal effect of imipramine on immobility time, our results clearly indicated that prenatal supra-therapeutic dose of buprenorphine induced depression-like phenotypes in their pups at postnatal day 21, independent of gender. Our results showed that depression-like changes were accompanied by alterations in several depression-related parameters, including elevation of oxidative stress, reduction of plasma levels of BDNF and serotonin, and attenuation of intracellular BDNF/TrkB-mediated signaling. Taken together, our results suggest that prenatal exposure to supra-therapeutic dose of buprenorphine might result in depression-like phenotypes associated with decreased BDNF action in weanlings.

Depression is a heterogeneous clinical disorder and its cause is not clear. The complexity of the disease is vast and involves several mechanisms, including disturbance of neurogenesis, genetic predisposition, deficiency of monoamines, hypercortisolemia, reduction of neurotrophins, inflammation, and oxidative stress [25], [31], [32]. Previous studies have linked increased oxidative stress and an impaired antioxidant system with mood disorders, including depression [24], [33]. Prenatal exposure to a supra-therapeutic dose of buprenorphine caused changes in the equilibrium between antioxidant and prooxidant activities, favoring the latter, by increasing production of brain MDA and oxidized GSH, and decreasing the reduced GSH and GPx activity. The increased oxidative stress after supra-therapeutic dose of buprenorphine exposure found in the present study was consistent with the results of a previous study that showed animals exposed to morphine exposure exhibited signs of oxidative stress in brains [34]. Based on these studies, oxidative stress appears to be a causative event for the development of depression-like phenotypes in pups born to dams exposed to supra-therapeutic dose of buprenorphine.

The neurotransmitter serotonin has long been implicated in the pathophysiology and treatment of mood disorders, particularly depression [26]. Beyond the serotonergic system, neurotrophins, especially BDNF, have been shown to promote neuronal survival, differentiation, function, and plasticity, suggesting that BDNF also plays a key role in the pathophysiology of depression [23]. In the present study, we observed an inhibitory effect induced by prenatal supra-therapeutic buprenorphine exposure in circulating levels of BDNF and serotonin. A growing number of clinical and experimental studies have demonstrated that depression is accompanied by decreased levels of BDNF and serotonin. Conversely, treatment with antidepressants increases BDNF expression, and brain infusion of BDNF produces antidepressant-like actions [23], [35], [36]. Based on these studies, our findings suggest that pups born to dams exposed to supra-therapeutic dose of buprenorphine show depression-like characteristics with decreased level of BDNF and serotonin.

It has been demonstrated that intracellular signaling molecules and transcription factors regulate the function of neurons and associated expression of neurotrophins and neurotransmitters. CREB is one of the most studied phosphorylation-activated transcription factors linked to depression, and its activation plays a critical role in inducing a transcription-dependent program for gene expression such as BDNF [22], [23]. The status of CREB phosphorylation at serine 133 is induced by multiple upstream regulators, including ERK and PKA [22], [27], [28]. Reduced expression and activity in ERK, PKA, and CREB have been seen in depressive suicide victims [37]–[39]. The inactivation of ERK, PKA, or CREB is capable of decreasing BDNF expression, producing depression-like phenotypes, and attenuating the actions of antidepressants. Parallel studies further demonstrate their activation in response to antidepressant treatment [21], [23], [28], [30]. Consistent with these findings, our data clearly showed a strong association among CREB signaling, BDNF expression, and depression-like neurobehaviors after prenatal supra-therapeutic buprenorphine exposure. In addition, this study provides evidence showing that among the multiple mechanisms involved in the development of depression-like neurobehavior, the inhibition of the CREB pathway, including downregulation of ERK phosphorylation, PKA activity, CREB phosphorylation, and CREB DNA-binding activity, might contribute in part to the decreased expression of BDNF and the resulting depression-like phenotypes.

Another interesting finding in this study was that the development of depression-like phenotypes after prenatal exposure to a supra-therapeutic dose of buprenorphine was accompanied by decreased circulating levels of BDNF and serotonin, TrkB phosphorylation, and CREB activity. The activation of CREB plays a crucial role in response to diverse signal transduction cascades activated by hormones, growth factors, synaptic activity, and other cellular stimuli implicated in neuronal plasticity. In addition to being a target of CREB, BDNF can itself recruit this particular transcription factor through BDNF/TrkB-mediated signaling by activating ERK, leading to the activation of CREB. Furthermore, the activation of the monoamine system could cause alterations of adenylyl cyclase activity via G proteins. cAMP elevation results in the activation of PKA [29], [40]. With regard to the therapeutic mechanism of action in antidepressant treatment, altered circulating levels of serotonin have been shown to be closely associated with depression [41]. These findings thus might set up a potential positive feedback loop between BDNF/serotonin expression and CREB activity. A direct consequence of decreased BDNF and serotonin expression and CREB activity could lead to a vicious cycle, further downregulating their expression and activity resulting in initiation and/or augmentation of depression-like neurobehaviors in pups born to dams prenatally exposed to a supra-therapeutic dose of buprenorphine.

Perinatal opioid exposure has effects in regulating the synthesis of neurotrophins and neurotransmitters, neurogenesis, and myelination, and is known to produce short- and long-term neurobehavioral changes in offspring [7], [9]–[15]. Although a prenatal supra-therapeutic dose of buprenorphine failed to cause severe maternal and fetal or neonatal mortality and morbidity, the application of a forced swimming test and tail suspension test revealed that pups born to dams with prenatal exposure to a supra-therapeutic dose of buprenorphine showed depression-like phenotypes at postnatal day 21, independent of gender. The depression-like phenotypes in pups were accompanied by elevation of oxidative stress, reduction of plasma levels of BDNF and serotonin, and attenuation of CREB signaling. The induction of oxidative stress, reduction of BDNF and serotonin, and attenuation of CREB signaling by prenatal exposure to a supra-therapeutic dose of buprenorphine provides evidence of a potential mechanism for the development of depression-like neurobehaviors. Despite the documented depression-like neurobehavioral effects of buprenorphine exposure, the opioid receptors involved and the potential long-term effects of neurobehavioral changes remain unclear and require further investigation. Another limitation in this study was that the biochemical analyses were done only in cortical tissues. The data of other tissues such as hippocampus will strengthen our study.
